# Supplementary figures and images for: Aqueous humour proteins and treatment outcomes of anti-VEGF therapy in neovascular age-related macular degeneration
Source: PLoS One. 2020 Mar 10;15(3):e0229342. doi: 10.1371/journal.pone.0229342 (PMC7064238; doi:10.1371/journal.pone.0229342)

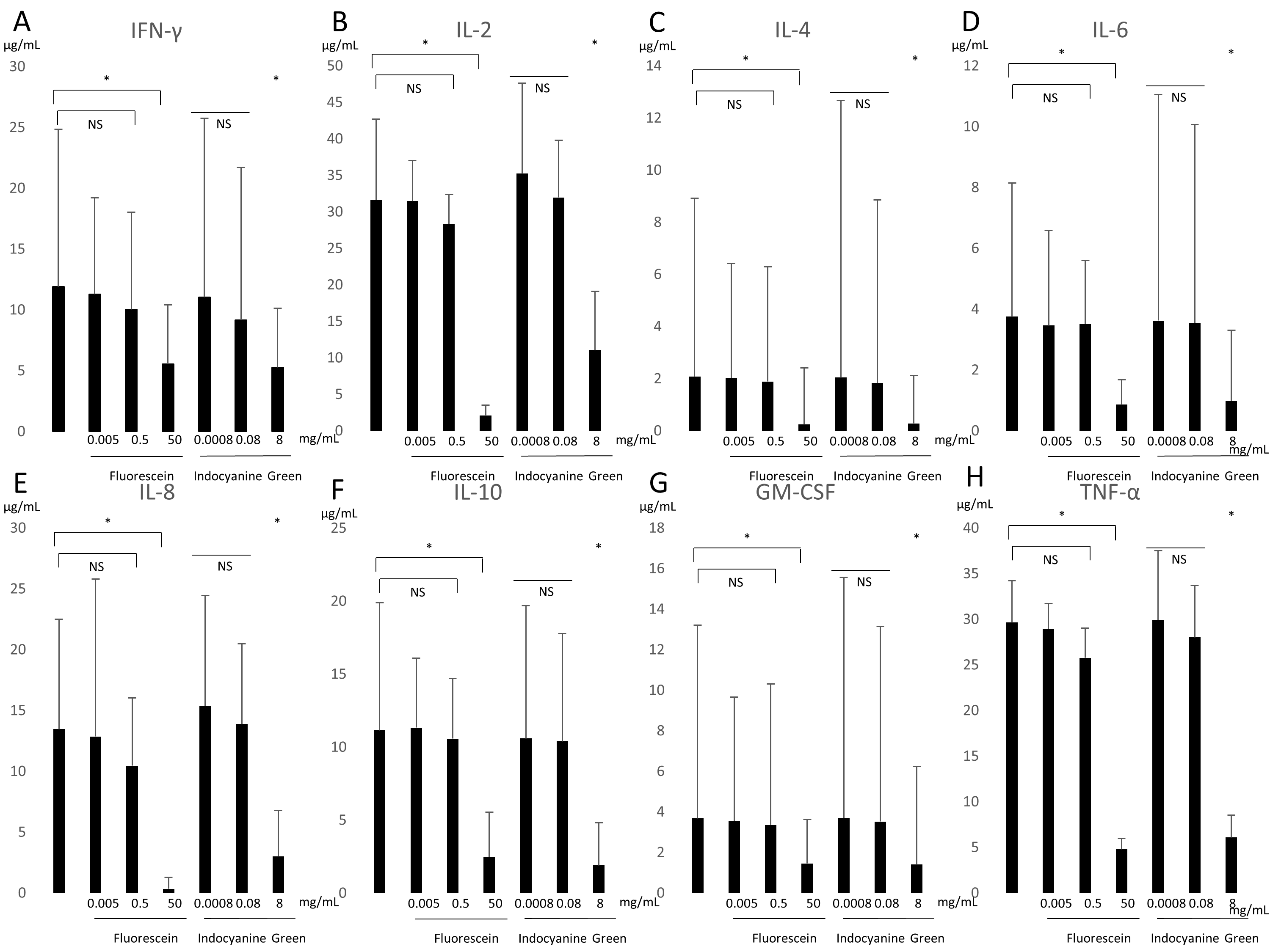

Supplement: S1 Fig — (TIF) [file pone.0229342.s001.tif]
