# Supplementary material for: Aqueous humour proteins and treatment outcomes of anti-VEGF therapy in neovascular age-related macular degeneration
Source: PLoS One. 2020 Mar 10;15(3):e0229342. doi: 10.1371/journal.pone.0229342 (PMC7064238; doi:10.1371/journal.pone.0229342)
Supplement: S1 Table — (DOCX) [file pone.0229342.s002.docx]

S1 Table. Factors associated with baseline characteristics

|  | CRT (µm) |  | CCT (µm) |  |
| --- | --- | --- | --- | --- |
|  | Univariate* | Multivariate | Univariate* | Multivariate |
| Age (years) | −0.018 (0.92) |  | −0.33 (0.078) | **−3.5 (-6.2 to 0.8, 0.016)*** |
| Sex (Male) | 13 (0.84) |  | 6.3 (0.80) |  |
| BCVA (logMAR) | 0.43 (0.020)* |  | 0.15 (0.42) |  |
| Disease type (PCV) | 30 (0.61) |  | 38 (0.11) |  |
| Duration of disease (months) | −0.11 (0.58) |  | 0.30 (0.11) | **2.8 (0.4 to 5.1, 0.027)*** |
| GLD (µm) | **0.40 (0.032)*** | **0.026 (0.003 to 0.048, 0.032)*** | 0.14 (0.48) |  |
| PVD (+) | −53 (0.37) |  | 8.2 (0.73) |  |
| Axial length (mm) | −0.24 (0.21) |  | −0.17 (0.37) |  |
| CRT (µm) |  |  | 0.071 (0.72) |  |
| CCT (µm) | 0.071 (0.72) |  |  |  |
| VEGF (pg/mL) | 0.25 (0.19) |  | −0.20 (0.30) |  |
| CXCL1 (pg/mL) | 0.26 (0.17) |  | −0.052 (0.79) |  |
| IP-10 (pg/mL) | **0.44 (0.016)*** |  | −0.083 (0.67) |  |
| CXCL12 (pg/mL) | 0.19 (0.33) |  | −0.090 (0.64) |  |
| CXCL13 (pg/mL) | 0.30 (0.11) |  | 0.067 (0.73) |  |
| MCP-1 (pg/mL) | **0.41 (0.029)*** |  | −0.16 (0.40) |  |
| CCL11 (pg/mL) | 0.082 (0.67) |  | −0.26 (0.18) |  |
| IL-6 (pg/mL) | **0.47 (0.010)*** | **82 (28 to 135, 0.0047)*** | −0.051 (0.79) |  |
| IL-10 (pg/mL) | 0.18 (0.35) |  | −0.12 (0.52) |  |
| MMP-9 (pg/mL) | −0.039 (0.84) |  | 0.063 (0.75) |  |
| R2 |  | 0.28 |  | 0.20 |

Univariate: Pearson's Correlation (for continuous variables) and Spearman's rho (for categorical variables). R (*P* value).

Multivariate analysis was performed after stepwise variable selection (BIC, forward method). β Coefficient (95% confidence interval, *P* value). *: *P* < 0.05
