# Supplementary material for: Aqueous humour proteins and treatment outcomes of anti-VEGF therapy in neovascular age-related macular degeneration
Source: PLoS One. 2020 Mar 10;15(3):e0229342. doi: 10.1371/journal.pone.0229342 (PMC7064238; doi:10.1371/journal.pone.0229342)
Supplement: S2 Table — (DOCX) [file pone.0229342.s003.docx]

|  | CRT change (µm) |  | CCT change (µm) |  |
| --- | --- | --- | --- | --- |
|  | Univariate* | Multivariate | Univariate* | Multivariate |
| Age (years) | -0.12 (0.53) | **-4.5 (-8.1 to -0.9, 0.020)*** | 0.36 (0.053) | **2.3 (0.6 to 4.1, 0.012)*** |
| Gender (Male) | 7.4 (0.98) |  | -1,8 (0.94) |  |
| BCVA (logMAR) | -0.33 (0.079) |  | -0.15 (0.43) |  |
| Disease type (PCV) | -86 (0.078) | **-38 (10 to 65, 0.010)*** | -43 (0.069) |  |
| Duration of disease (months) | 0.10 (0.60) |  | -0.26 (0.18) |  |
| GLD (µm) | -0.22 (0.26) | 0.013 (-0.001 to 0.026, 0.068) | -0.32 (0.096) | **-0.0090 (-0.153 to -0.0028, 0.0069)*** |
| PVD (+) | 33 (0.51) |  | 7.9 (0.74) |  |
| Axial length (mm) | 0.25 (0.19) |  | 0.094 (0.63) |  |
| CRT (µm) | **-0.75 (<0.001)*** | **-0.65 (-0.80 to -0.50, <0.001)*** | -0.047 (0.81) | **0.12 (0.04 to 0.19, 0.0037)*** |
| CCT (µm) | 0.060 (0.76) |  | **-0.74 (<0.001)*** | **-0.69 (-0.86 to -0.52, <0.001)*** |
| VEGF (pg/mL) | -0.21 (0.27) |  | 0.10 (0.59) |  |
| CXCL1 (pg/mL) | -0.29 (0.12) |  | 0.016 (0.93) |  |
| IP-10 (pg/mL) | **-0.45 (0.014)*** |  | -0.20 (0.30) |  |
| CXCL12 (pg/mL) | -0.34 (0.075) | -37 (-77 to 3, 0.076) | 0.013 (0.95) |  |
| CXCL13 (pg/mL) | -0.32 (0.087) |  | -0.20 (0.29) |  |
| MCP-1 (pg/mL) | **-0.45 (0.014)*** |  | -0.055 (0.78) |  |
| CCL11 (pg/mL) | -0.21 (0.26) |  | 0.16 (0.42) |  |
| IL-6 (pg/mL) | **-0.44 (0.016)*** |  | -0.26 (0.17) | **-34 (-49 to -19, <0.001)*** |
| IL-10 (pg/mL) | -0.072 (0.71) |  | 0.084 (0.66) |  |
| MMP-9 (pg/mL) | 0.21 (0.28) | **24 (4 to 44, 0.024)*** | 0.070 (0.72) |  |
| R square |  | 0.73 |  | 0.75 |

S2 Table. Factors associated with CRT and CCT changes at 2 months

Univariate: Pearson's correlation (for continuous variables) and Spearman's rho (for categorical variables). R (*P* value).

Multivariate analysis was performed after stepwise variable selection (BIC, forward method). β Coefficient (95% confidence interval, *P* value). **P* < 0.05.
