# Supplementary material for: Aqueous humour proteins and treatment outcomes of anti-VEGF therapy in neovascular age-related macular degeneration
Source: PLoS One. 2020 Mar 10;15(3):e0229342. doi: 10.1371/journal.pone.0229342 (PMC7064238; doi:10.1371/journal.pone.0229342)
Supplement: S1 Text — (DOCX) [file pone.0229342.s004.docx]

**S1 Text. Supplementary Information of Central Retinal Thickness and Central Choroidal Thickness**

**Central retinal thickness**

In univariate analysis, increased CRT was significantly associated with large GLD (r = 0.40, *P* = 0.032), high IP-10 (r = 0.44, *P* = 0.016), high MCP-1 (r = 0.41, *P* = 0.029), and high IL-6 (r = 0.47, *P* = 0.010). Among these, GLD (β coefficient = 0.026, *P* = 0.032) and IL-6 (β coefficient = 82, *P* = 0.0047) were selected and remained significant in multivariate analysis after stepwise variable selection. (Supplementary Table S1) Similarly, we assessed CRT change at 2 months using stepwise variable selection. The results demonstrated better CRT change at 2 months was associated with old age (β coefficient = −4.5, *P* = 0.020), disease type (PCV) (β coefficient = −38, *P* = 0.010), baseline thicker CRT (β coefficient = −0.65, *P* < 0.001), and lower MMP-9 (β coefficient = 24, *P* = 0.024). (S1 Table)

**Central choroidal thickness**

Regarding CCT, in univariate analysis, baseline CCT was not significantly associated with any variables. Stepwise variable selection selected young age (β coefficient = −3.5, *P* = 0.016) and long duration of disease (β coefficient = 2.8, *P* = 0.027) as factors associated with baseline thicker CCT (Supplementary Table 1). Larger CCT reduction at 2 months was associated with young age (β coefficient = 2.3, *P* = 0.012), large GLD (β coefficient = −0.0090, *P* = 0.0069), baseline thinner CRT (β coefficient = 0.12, *P* = 0.0037), baseline thicker CCT (β coefficient = −0.69, *P* < 0.001), and higher IL-6 (β coefficient = −34, *P* < 0.001). (S2 Table)
