# Supplementary material for: Aqueous humour proteins and treatment outcomes of anti-VEGF therapy in neovascular age-related macular degeneration
Source: PLoS One. 2020 Mar 10;15(3):e0229342. doi: 10.1371/journal.pone.0229342 (PMC7064238; doi:10.1371/journal.pone.0229342)
Supplement: S2 Text — (DOCX) [file pone.0229342.s005.docx]

**S2 Text. Influence of Fluorescein and Indocyanine Green to Measurements using Multiplex Cytokine Assay**

**IFN-γ, IL-2, IL-4, IL-6, IL-8, IL-10, GM-CSF, and TNF-α Concentrations with Various Concentration of Fluorescein and Indocyanine Green using Multiplex Cytokine Assay**

%CV of multiplex cytokine assay kit (Bio-Plex Pro™ Human Cytokine Grp l Panel 8-Plex) was less than 10. We set effect size d = 1.2, α err prob = 0.05, power (1 – β err prob) = 0.8. Total sample size was calculated as 20 using G*Power 3.1.9.4. Measurements were done according to the text of the manufacture. 1/2048 diluted kit standard 0 were used as control. Control and control with 0.005, 0.5, and 5 mg/dL fluorescein and 0.0008, 0.08, 8 mg/dL of indocyanine green were measured. Each n = 10. There was no significance between control and each control with 0.005 and 0.5 mg/dL fluorescein and 0.0008 and 0.08 mg/dL of indocyanine green (double tailed student’s *t*-test). Control with 5 mg/dL fluorescein and control with 8 mg/dL indocyanine green were measured lower than control (*P* < 0.05). Assuming a total blood volume of 5 L, the concentrations of fluorescein and indocyanine green diluted in whole blood were 0.1 mg/mL and 0.005 mg/mL, and it was considered that measurement inhibition did not occur in aqueous humor. A: IFN-γ. B: IL-2. C: IL-4. D: IL-6. E: IL-8. F: IL-10. G: GM-CSF. H: TNF-α. (S1 Figure)
