# Supplementary material for: Aqueous humour proteins and treatment outcomes of anti-VEGF therapy in neovascular age-related macular degeneration
Source: PLoS One. 2020 Mar 10;15(3):e0229342. doi: 10.1371/journal.pone.0229342 (PMC7064238; doi:10.1371/journal.pone.0229342)
Supplement: Attachment — Submitted filename: Response to Reviewers.docx [file pone.0229342.s007.docx]

We would appreciate receiving your revised manuscript by Feb 02 2020 11:59PM.

Dear editor and reviewers,

Thank you for reviewing our manuscript for its submission to PLOS ONE. We hereby submit a revised version of the manuscript that addresses the point-by-point response raised during the review process.

Reviewer #1: I do congratulate the authors for their job improving the manuscript, which is now technically sound and expressively strong. I can only point out two minor questions to undertake.

Lines 118-120: clarify whether VEGF in these 29 samples was vitreous or aqueous humour. Moreover, since not all samples were analyzed for VEGF search (as stated in your response), it ends up behaving as a huge bias for the study. Some could even think that the authors choose which samples they finally analyzed. I feel it is not enough to state a reference to back up such reasoning and I would rather include a detailed explanation, like the one that has been provided in the response, in the manuscript.

**First, these 29 samples were aqueous humour. Second, we have added explanations as you suggested. (lines 127-129)**

Conclusion: lacks a statement on IL-10 at 2 months associated with the number of injections, which has been extensively tackled in the Discussion, and should be at least briefly commented here.

**We have updated the conclusion section in accordance with your comment. (lines 380-381)**

Reviewer #3: In this paper Arai et al. investigated the relationship between Aqueous humour proteins and anti-VEGF therapy outcomes in nAMD. The authors have addressed previous comments. I have two additional minor comments:

1. Abstract: The authors state in the final sentence that "In conclusion, aqueous humour protein concentrations may be good predictors of BCVA change over 12 months and the number of injections in pro re nata treatment of exudative nAMD.". This is not my impression from this study - this study can only conclude that aqueous humor proteins may have predictive abilities - to determine whether they are good predictors, further biomarker analyses need to be performed.

**We have updated the abstract section in accordance with your comment. (line 45)**

2. Regarding the limitations of this study. This is perhaps in the lines of my comment #1. A limitation of this study is the lack of another independent sample, wherein the potentially predictive abilities of aqueous humor proteins can be tested. This approach allows concluding whether these proteins truly possess predictive abilities and how good they are in doing so.

**We agree with this. We have revised the limitation section. (lines 369-370)**

3. Regarding methods: To my knowledge, the Procarta® Cytokine Assay Kit is a multiplex cytokine assay that is read using a fluorescence-based method. Do you have any insight from a pilot study or any investigation into whether fluorescein or indocyanine green (which is used in the retinal angiography in relation to diagnosis) influences measurements obtained from the Procarta® Cytokine Assay Kit? If not, then I think it would be worth mentioning that this is a potential source of bias and thus a limitation that must be kept in mind when interpreting the results of this study. Also, it is unclear in the manuscript when you did the sampling. Immediately after the diagnosis? Immediately prior to the injection? Immediately after the injection? Was it prior to the injection in baseline, but after at the first follow-up? These factors are important details with potential influence to the results. These factors should be outlined in a clear fashion.

**We performed a preliminary test, and confirmed that the fluorescein and indocyanine green did not affect the measurement of cytokine levels in this Cytokine Assay Kit. We have added the results as S2-text for your review. In the text we mentioned “dye angiography did not affect the measurements (data not shown).” (lines 123 to 124)**
